# Biasing neural network dynamics using non-invasive brain stimulation

**DOI:** 10.3389/fnsys.2014.00246

**Published:** 2015-01-12

**Authors:** Martijn E. Wokke, Lotte J. Talsma, Marlies E. Vissers

**Affiliations:** ^1^Amsterdam Brain and Cognition, University of AmsterdamAmsterdam, Netherlands; ^2^Consciousness, Cognition and Computation Group, Department of Psychology, Université Libre de BruxellesBrussels, Belgium; ^3^Department of Psychology, University of AmsterdamAmsterdam, Netherlands

**Keywords:** performance enhancement, neural networks, neuromodulation, TMS, tDCS

## Abstract

Recently, non-invasive brain stimulation (NBS) has been discovered as a tool to improve human performance on a wide variety of tasks. Although these observations are highly intriguing, the underlying mechanisms of such enhancements are still poorly understood. Here, we argue that in order to advance our understanding of these mechanisms it is necessary to focus on intrinsic network dynamics in the brain. Taking into account well-known network dynamics, increased excitation in one particular network or brain region may necessarily lead to inhibition of an opposing network (and vice versa). As a consequence, observed behavioral improvements due to NBS may emerge from a shift in the balance between (competing) neural networks in the brain, implicating that behavioral enhancement due to stimulation most likely comes with a cost or side effect. We conclude that more elaborate experimental designs are essential for a better understanding of the relationship between network interactions and the behavioral effects of NBS.

## Introduction

In the last decade the use of non-invasive brain stimulation (NBS) techniques in cognitive neuroscience has grown explosively. Especially transcranial magnetic stimulation (TMS) and transcranial direct current stimulation (tDCS) have proven to be fruitful tools to causally link a wide range of brain regions or neural networks to perception, motor action and higher-level cognition. Moreover, NBS has been welcomed enthusiastically as a method to improve various aspects of human behavior (Luber and Lisanby, 2014). Yet, the full scope and range of the effects of NBS are currently poorly understood. In order to gain a more thorough understanding of the effects caused by stimulation, we will advocate that the observed effects of NBS on brain functioning should be seen in the light of complex interplays between task-relevant and task-irrelevant neural networks in the brain.

Almost three decades ago, TMS was introduced by Barker and colleagues (Barker et al., 1985) as a NBS technique that was able to safely affect brain function in humans (see Rossi et al., 2009). The effect of TMS is based on the principle of electromagnetic induction, in which a rapidly changing magnetic field induces a current in an electrically conducting medium, such as neural tissue. When TMS currents meet the right requirements (e.g., amplitude, duration and frequency, see Wagner et al., 2009) neural function and behavior can be altered, even outlasting the period of stimulation. The application of tDCS in cognitive neuroscientific research was introduced several years later than TMS (Priori et al., 1998; Nitsche and Paulus, 2000), but the popularity of tDCS as a neuromodulatory tool led to a rapidly growing body of research on the effects of tDCS on perception, action and cognition. tDCS can modulate cortical excitability of neural activity by the induction of weak anodal and cathodal electrical currents flowing though the cerebral cortex. The polarity is of great influence on the neuromodulatory effect of tDCS: Where anodal (positive polarization) stimulation in general facilitates cortical excitability of the underlying tissue, cathodal stimulation increases the threshold for neuronal firing (Liebetanz et al., 2002; Krause et al., 2013; however, note that the effect of polarity on neuronal firing may also depend on the state of the targeted brain region during stimulation, see e.g., Krause and Cohen Kadosh, 2014).

Initially, NBS was used to determine whether modulation of neural activity in a particular brain region was able to disrupt performance associated with normal network functioning. However, various studies began to demonstrate that tempering normal network functioning could in fact result in paradoxical improvements of performance (Luber and Lisanby, 2014). Recently, the rise of tDCS and new TMS protocols has highlighted the potential of NBS as a technique that can be used to improve brain functioning in healthy individuals or in patients suffering from neurological or psychiatric illness (Coffman et al., 2014). Although these findings are very intriguing and seem promising for both healthy and clinical populations, the underlying neural mechanisms subserving the augmentation of brain function following TMS and tDCS remains largely elusive.

In this paper we will exclusively focus on two factors that are important when considering the potential benefits and costs of neurostimulation techniques. These factors are not directly related to parameters (e.g., polarity or frequency) in *generating* a stimulation effect (for an extensive discussion of stimulation parameters in this respect, see e.g., Walsh et al., 2003; Flöel, 2014), but concern a conceptual framework for studying NBS. Firstly, there is ample evidence on facilitatory and inhibitory interactions between different functional networks in the brain (Kinsbourne, 1987; Calautti and Baron, 2003; Fox et al., 2005; Szczepanski and Kastner, 2013), stressing the importance of reckoning the brain as a complex constellation of functional networks. This notion is already widely self-evident in certain domains, such as in research on brain connectivity (e.g., Sporns, 2013) and rehabilitation (Calautti and Baron, 2003), however it currently seems to have little influence in the design and interpretation of NBS experiments and effects. Whereas it is conceivable that stimulation of one particular network or brain region may lead to cognitive benefits that come at the cost of other cognitive processes, studies reporting brain function enhancement typically focus on the effects of stimulation on the targeted process or ability, without co-assessing possible unintended effects on other functions. However, by selectively studying intended behavioral improvements, it is conceivable that cognitive enhancements are commanding the spotlights, while potential costs keep on dancing in the dark. As a consequence, current approaches obscure the value of observed enhancement effects in a broader sense. Secondly, the effects of TMS and tDCS have been shown to be dependent on the state of the probed network (Silvanto et al., 2007; Krause and Cohen Kadosh, 2014). The specific context in which stimulation is applied seems to be critical for the behavioral effect, demonstrating how observed enhancements are emerging from complex, dynamic interactions between internal and external modulations of network activity.

We will first address a small selection of studies demonstrating enhancement of performance by TMS and tDCS in the sensory and higher-level cognitive domain. We will discuss these findings in light of brain network interactions and the modulatory role of the functional state of the stimulated network, in order to illustrate the importance of taking network interactions into account when studying the effects of NBS on behavior.

## Enhancement of visual perception

One of the first studies demonstrating enhancement of sensory processing induced by TMS manipulated visual attention by applying repetitive transcranial magnetic stimulation (rTMS) to the human parietal cortex (Hilgetag et al., 2001). Previously, a great deal of what was known about the neural mechanism of visual attention stemmed from patients suffering from visual neglect. Visual neglect is typically caused by lesions to the posterior parietal (or frontal) cortex, resulting in a deficit in the ability to draw attention towards the visual space contralateral to the lesion. This pattern of findings led to a model of cross-hemispheric competition (Kinsbourne, 1987; Szczepanski and Kastner, 2013), in which the balance in inter-hemispheric distribution of attentional resources is maintained via mutual inhibition. Interestingly, while researchers are typically focused on contralateral attentional deficits accompanying visual neglect Hilgetag et al. (2001) demonstrated that the induction of a “virtual lesion” to the parietal cortex could actually result in increased ipsilateral visual attention. These findings demonstrate how the intrinsic balance between neural activity across hemispheres is essential for typical perceptual functioning, and how disturbance of this balance can lead to perceptual deficits such as neglect.

**Figure 1:**
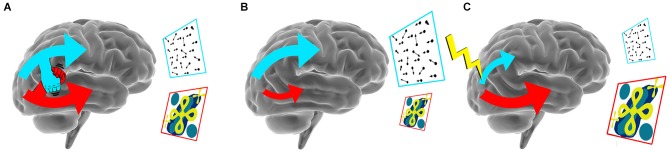
**Biasing the balance between networks with NBS. (A)** A schematic illustration of competing network interactions in the brain during a motion discrimination (blue frame) and a shape discrimination (red frame) task, that both rely on different brain networks. **(B)** When engaging in a motion discrimination task activity in the dorsal network increases, while activity in the ventral stream decreases. **(C)** Disruption of dorsal (visual) activity results in decreased performance in motion direction discrimination while decreased dorsal competition results in enhanced performance on the shape discrimination task.

Disruption of more subtle network interactions than the inter-hemispheric interplay discussed above has also been found to affect behavioral performance. This was illustrated in a TMS experiment on visual feature processing (Walsh et al., 1998). Walsh et al. found that disruption of cortical area HMT+/V5, a key region involved in motion processing, impaired performance during a visual search task when motion was the critical target feature. In contrast, disruption of HMT+/V5 resulted in an initially unexpected enhancement of performance when color or form were the essential features of the target. The enhancement of performance caused by the disruption of HMT+/V5 was unexpected, as TMS was considered to induce neural noise, and thereby, to deteriorate normal behavior. The unexpected enhancement was interpreted to be the result of the temporarily reduced competition of (visual) brain areas for limited processing resources, such as energy and communication with other brain regions. Thus, disruption of HMT+/V5 by TMS likely shifted the balance of neural resources towards other visual areas, improving visual perception for features processed in these other brain regions. Recently, we observed additional supporting evidence for a model in which brain regions engage in a “battle for resources” (Wokke et al., 2014). In two experiments, we probed the role of area HMT+/V5 and the object sensitive lateral occipital region (LO) during a figure discrimination task that dominantly relied on HMT+/V5 processing. Disruption of activity in LO and HMT+/V5 led to opposing effects on performance, depending on the stimulation site. Disruption of HMT+/V5 resulted in decreased discriminability, whereas participants’ discriminability improved when activity in LO was perturbed. Complementary to the findings by Walsh et al. (1998), we demonstrated that the workings of HMT+/V5 improved during a motion-defined figure discrimination task when we disrupted a cortical region specialized in task-irrelevant properties. These findings provide converging evidence for competitive interactions between extrastriate cortical areas. Such observations of improved performance due to reduced neural activity in task-irrelevant regions, a phenomenon that has been dubbed “addition-by-subtraction” (Luber and Lisanby, 2014), are strong examples of the notion that effects of NBS are established due to the intrinsic interactions of functional brain networks (see Figure 1).

## Improved cognitive performance

The growing popularity of tDCS has led to a steep rise in the number of studies on enhancement of cognitive functions such as attention, (motor) learning, working memory, and even complex problem solving (Fregni et al., 2005; Cohen Kadosh et al., 2010; Chi and Snyder, 2012; Coffman et al., 2012). For example, there is evidence for a positive effect of tDCS over frontal brain regions on working memory performance. One of the first studies on the effects of tDCS on higher-order cognition used anodal tDCS over left prefrontal dorsolateral cortex (lDLPFC) to study its effects on working memory performance (e.g., Fregni et al., 2005). Anodal tDCS over lDLPFC was shown to reduce the number of errors people make on a 3-back working memory task, increasing accuracy of performance. However, there are also studies that fail to demonstrate a positive effect of tDCS on working memory functioning (for a review, see Coffman et al., 2014), complicating the interpretation of observed enhancement effects.

Recently, an elegant study by Iuculano and Cohen Kadosh (2013) revealed detrimental as well as beneficial effects of tDCS on cognition. In this study, participants performed a mathematical training during which tDCS was applied on different sites. Part of the participants received tDCS over posterior parietal cortex (PPC), whereas other participants received stimulation over DLPFC. Results showed that stimulation of the PPC led to increased speed of learning, but to impaired automaticity for the learned materials, whereas the opposite was found to be the case for stimulation over DLPFC. Thus, these findings show that stimulating the brain at different locations may have positive but also disruptive effects on cognitive performance. As suggested by the authors themselves, the eventual benefits and costs that come with a particular stimulation method may be due to corticocortical interactions, and induced shifts between metabolic prioritization. Clearly, this study illustrates the importance of broadening the scope of investigated abilities in order to detect whether behavioral improvements in one domain may come at the expense of another, emphasizing the importance of taking network configurations into account when assessing behavioral effects due to NBS. However, whereas the study by Iuculano and Cohen Kadosh (2013) was directed at comparing stimulation effects on different aspects of a targeted cognitive function (mathematical learning), it would be desirable to pursue this type of research using designs in which one single site is being stimulated, while multiple (cognitive) functions are investigated. This approach would allow one to investigate the extent to which collateral impairments in behavior arise due to intrinsic interactions between functional networks in the brain.

Another important factor to take into account is the state of a network during stimulation (Krause and Cohen Kadosh, 2014). It seems that the initial neural activation state during stimulation determines the behavioral effect of stimulation (Silvanto et al., 2008). When stimulation is applied during performance of a task, its effects have been shown to differ from stimulation during rest (Andrews et al., 2011). Another interesting finding concerning the effect of cognitive state is that application of tDCS over lateral PFC when subjects are not involved in a cognitive task, modulates activity in task-positive as well as task-negative networks (as measured with fMRI; Keeser et al., 2011). Thus, although stimulation effects depend on the currently dominant functional network, these findings show that stimulation effects are not restricted to the currently active network. Therefore, full comprehension of the potential scope of NBS requires a more complete understanding of the extent to which stimulation has an effect on targeted, as well as task-irrelevant and non-targeted networks.

## Opposing network dynamics

In the last decade a strong interest in competing network dynamics has been sparked by observed relations between opposed activity levels in task-positive (e.g., attention, fronto-parietal) and task-negative (e.g., default mode) networks and performance on a variety of tasks (Raichle et al., 2001; Fox et al., 2005; Weissman et al., 2006; Kelly et al., 2008; Hampson et al., 2010). Crucially, there seems to be a competitive balance of activity between task-positive and task-negative networks during task performance (Wojciulik and Kanwisher, 1999; McKiernan et al., 2003). During task performance activity increases in regions that are supporting task execution, whereas activity decreases in regions associated with task irrelevant (or task-opposing) processes. A growing amount of studies demonstrate the existence of strong anti-correlations between task-negative networks and task-positive networks (e.g., Fox et al., 2005). These anti-correlations have been shown to relate to performance, such that stronger anti-correlations are predictive of better cognitive performance (Kelly et al., 2008; Hampson et al., 2010). The dichotomy in activity levels observed in different networks during task performance has been suggested to be an intrinsic property of the organization of the brain (Fox et al., 2005).

It is conceivable that an external modulation of the competition between antagonistic networks in the brain could be beneficial in certain circumstances. For instance, in people with disorders such as autism spectrum disorder (ASD), anti-correlations between the default mode network and task-positive regions were found to be less pronounced than in typical subjects (Kennedy et al., 2006). In addition, decreased anti-correlations has been related to ASD symptoms (Anderson et al., 2011). These patterns demonstrate possible detrimental effects of atypical competition between different networks. Interestingly, Josipovic et al. (2012) recently demonstrated that anti-correlations between task-positive and task-negative networks could be differentially modulated depending of the cognitive style during meditation, thus disregarding them as an immutable characteristic of the organization of the brain. Further, the balance between competing networks might be adjusted dynamically to fit currently relevant behavioral goals by regulatory networks involved in top-down control (Spreng et al., 2013). Thus, whereas the interrelatedness of networks might be an inherent property of the functional organization of the brain, the balance between different network states is likely to be flexible and sensitive to top-down control. In light of these findings, NBS could be instrumental by altering the strength of competing interactions between different networks, or dichotomously increase and decrease the amount of synchrony within each network (Peña-Gómez et al., 2012).

Based on findings revealing the dynamic competition between activated and deactivated networks and their effects on behavior, it seems evident that interfering with activity in one network by applying TMS or tDCS necessarily shifts the balance between task relevant and task-irrelevant networks. Taken together with the established functional links between network anti-correlations and healthy and efficient cognitive functioning, this induced balance shift between networks is likely to have an effect on cognitive performance. Therefore, an important venue for future research employing NBS is to examine to what extent the behavioral effects of these methods can be explained by a shift in the balance of activation between different networks, rather than the current approach of solemnly focusing on altered levels of activity within one network. The notion that the balance between competing networks is an important factor may also help to explain the importance of the state of a network during application of tDCS (McKiernan et al., 2003). When taking the interconnectedness of brain networks into account, it becomes trivial that effects of brain stimulation during a state in which different functional networks are anti-correlated differs from stimulation during rest, because of the large differences in the configuration of functional networks (Silvanto et al., 2008).

Furthermore, the principle of improving one cognitive function by suppression of task-irrelevant neural processing, which was observed in TMS research described above, is possibly also at play during application of tDCS. This principle can be understood in terms of the framework proposed by Brem et al. (2014), which holds that the brain functions as a closed energy system. Under this assumption, brain stimulation would always have a “net zero-sum” effect, meaning that enhancement of one domain or function will always have costs for another domain or function. When the brain is stimulated, the distribution of resources over different brain regions or networks is externally modulated. Enhancement or suppression of activity in one neural network will necessarily lead to impairment or enhancement in another network, due to redistribution of available resources. At present we do not know whether brain stimulation exerts its effects by a temporary redistribution of resources, or whether it induces a different type of redistribution in the brain. In order to acquire more insight into the effects of NBS of distribution of neural resources, research on NBS should take the interrelatedness of different network dynamics as a central starting point.

## Conclusions

In the present paper we argued that recent findings of enhancement of brain function should be seen in light of a manipulation of the balance between different functional brain networks, which can result in improved behavior when applied in the right context. However, assuming NBS is biasing the brain towards one particular functional state, this might also have detrimental effects on performance, for example, by hampering flexible transitions between functional networks. It might therefore be misleading to speak of an *enhancement* of brain function, and, alternatively, it might be more appropriate to describe the NBS effects in terms of a *bias shift*.

More research on brain-wide effects of NBS will contribute to an understanding of the way the entire brain is affected by NBS at specific sites, and may yield ways to apply these techniques in more efficient ways. In addition, with a more thorough understanding of NBS effects, brain stimulation should become more applicable outside the realm of specific lab settings. Therefore, in order to move the field of NBS and its (clinical) applications forward, it is essential to extend current studies on the effects of NBS towards investigating the effects of stimulation on task-relevant as well as task-irrelevant functional brain networks.

## Conflict of interest statement

The authors declare that the research was conducted in the absence of any commercial or financial relationships that could be construed as a potential conflict of interest.

## References

[B1] Anderson J. S. Nielsen J. A. Froehlich A. L. DuBray M. B. Druzgal T. J. Cariello A. N.. (2011). Functional connectivity magnetic resonance imaging classification of autism. Brain 134, 3742–3754. 10.1093/brain/awr263 22006979 PMC3235557

[B2] Andrews S. C. Hoy K. E. Enticott P. G. Daskalakis Z. J. Fitzgerald P. B. (2011). Improving working memory: the effect of combining cognitive activity and anodal transcranial direct current stimulation to the left dorsolateral prefrontal cortex. Brain Stimul. 4, 84–89. 10.1016/j.brs.2010.06.004 21511208

[B3] Barker A. T. Jalinous R. Freeston I. L. (1985). Non-invasive magnetic stimulation of human motor cortex. The Lancet 325, 1106–1107. 10.1016/s0140-6736(85)92413-4 2860322

[B4] Brem A. K. Fried P. J. Horvath J. C. Robertson E. M. Pascual-Leone A. (2014). Is neuroenhancement by noninvasive brain stimulation a net zero-sum proposition?. Neuroimage 85, 1058–1068. 10.1016/j.neuroimage.2013.07.038 23880500 PMC4392930

[B5] Calautti C. Baron J. C. (2003). Functional neuroimaging studies of motor recovery after stroke in adults a review. Stroke 34, 1553–1566. 10.1161/01.str.0000071761.36075.a6 12738893

[B6] Chi R. P. Snyder A. W. (2012). Brain stimulation enables the solution of an inherently difficult problem. Neurosci. Lett. 515, 121–124. 10.1016/j.neulet.2012.03.012 22440856

[B7] Coffman B. A. Clark V. P. Parasuraman R. (2014). Battery powered thought: enhancement of attention, learning and memory in healthy adults using transcranial direct current stimulation. Neuroimage 85, 895–908. 10.1016/j.neuroimage.2013.07.083 23933040

[B8] Coffman B. A. Trumbo M. C. Clark V. P. (2012). Enhancement of object detection with transcranial direct current stimulation is associated with increased attention. BMC Neurosci. 13:108. 10.1186/1471-2202-13-108 22963503 PMC3494452

[B9] Cohen Kadosh R. Soskic S. Iuculano T. Kanai R. Walsh V. (2010). Modulating neuronal activity produces specific and long-lasting changes in numerical competence. Current Biol. 20, 2016–2020. 10.1016/j.cub.2010.10.007 21055945 PMC2990865

[B40] Flöel A. (2014). tDCS-enhanced motor and cognitive function in neurological diseases. Neuroimage 85, 934–947. 10.1016/j.neuroimage.2013.05.098 23727025

[B10] Fox M. D. Snyder A. Z. Vincent J. L. Corbetta M. Van Essen D. C. Raichle M. E. (2005). The human brain is intrinsically organized into dynamic, anticorrelated functional networks. Proc. Natl. Acad. Sci. U S A 102, 9673–9678. 10.1073/pnas.0504136102 15976020 PMC1157105

[B11] Fregni F. Boggio P. S. Nitsche M. Bermpohl F. Antal A. Feredoes E.. (2005). Anodal transcranial direct current stimulation of prefrontal cortex enhances working memory. Exp. Brain Res. 166, 23–30. 10.1007/s00221-005-2334-6 15999258

[B12] Hampson M. Driesen N. Roth J. K. Gore J. C. Constable R. T. (2010). Functional connectivity between task-positive and task-negative brain areas and its relation to working memory performance. Magn. Reson. Imaging 28, 1051–1057. 10.1016/j.mri.2010.03.021 20409665 PMC2936669

[B13] Hilgetag C. C. Théoret H. Pascual-Leone A. (2001). Enhanced visual spatial attention ipsilateral to rTMS-induced ‘virtual lesions’ of human parietal cortex. Nat. Neurosci. 4, 953–957. 10.1038/nn0901-953 11528429

[B14] Iuculano T. Cohen Kadosh R. (2013). The mental cost of cognitive enhancement. J. Neurosci. 33, 4482–4486. 10.1523/jneurosci.4927-12.2013 23467363 PMC3672974

[B15] Josipovic Z. Dinstein I. Weber J. Heeger D. J. (2012). Influence of meditation on anti-correlated networks in the brain. Front. Hum. Neurosci. 5:183. 10.3389/fnhum.2011.00183 22287947 PMC3250078

[B16] Keeser D. Meindl T. Bor J. Palm U. Pogarell O. Mulert C.. (2011). Prefrontal transcranial direct current stimulation changes connectivity of resting-state networks during fMRI. J. Neurosci. 31, 15284–15293. 10.1523/jneurosci.0542-11.2011 22031874 PMC6703525

[B17] Kelly A. M. Uddin L. Q. Biswal B. B. Castellanos F. X. Milham M. P. (2008). Competition between functional brain networks mediates behavioral variability. Neuroimage 39, 527–537. 10.1016/j.neuroimage.2007.08.008 17919929

[B18] Kennedy D. P. Redcay E. Courchesne E. (2006). Failing to deactivate: resting functional abnormalities in autism. Proc. Natl. Acad. Sci. U S A 103, 8275–8280. 10.1073/pnas.0600674103 16702548 PMC1472462

[B19] Kinsbourne M. (1987). Mechanisms of unilateral neglect. Adv. Psychol. 45, 69–86 10.1016/s0166-4115(08)61709-4

[B20] Krause B. Cohen Kadosh R. (2014). Not all brains are created equal: the relevance of individual differences in responsiveness to transcranial electrical stimulation. Front. Syst. Neurosci. 8:25. 10.3389/fnsys.2014.00025 24605090 PMC3932631

[B21] Krause B. Márquez-Ruiz J. Kadosh R. C. (2013). The effect of transcranial direct current stimulation: a role for cortical excitation/inhibition balance?. Front. Hum. Neurosci. 7:602. 10.3389/fnhum.2013.00602 24068995 PMC3781319

[B22] Liebetanz D. Nitsche M. A. Tergau F. Paulus W. (2002). Pharmacological approach to the mechanisms of transcranial DC-stimulation-induced after-effects of human motor cortex excitability. Brain 125, 2238–2247. 10.1093/brain/awf238 12244081

[B23] Luber B. Lisanby S. H. (2014). Enhancement of human cognitive performance using transcranial magnetic stimulation (TMS). Neuroimage 85, 961–970. 10.1016/j.neuroimage.2013.06.007 23770409 PMC4083569

[B24] McKiernan K. A. Kaufman J. N. Kucera-Thompson J. Binder J. R. (2003). A parametric manipulation of factors affecting task-induced deactivation in functional neuroimaging. J. Cogn. Neurosci. 15, 394–408. 10.1162/089892903321593117 12729491

[B25] Nitsche M. A. Paulus W. (2000). Excitability changes induced in the human motor cortex by weak transcranial direct current stimulation. J. Physiol. 527, 633–639. 10.1111/j.1469-7793.2000.t01-1-00633.x 10990547 PMC2270099

[B26] Peña-Gómez C. Sala-Lonch R. Junqué C. Clemente I. C. Vidal D. Bargalló N.. (2012). Modulation of large-scale brain networks by transcranial direct current stimulation evidenced by resting-state functional MRI. Brain Stimul. 5, 252–263. 10.1016/j.brs.2011.08.006 21962981 PMC3589751

[B27] Priori A. Berardelli A. Rona S. Accornero N. Manfredi M. (1998). Polarization of the human motor cortex through the scalp. Neuroreport 9, 2257–2260. 9694210 10.1097/00001756-199807130-00020

[B28] Raichle M. E. MacLeod A. M. Snyder A. Z. Powers W. J. Gusnard D. A. Shulman G. L. (2001). A default mode of brain function. Proc. Natl. Acad. Sci. U S A 98, 676–682. 10.1073/pnas.98.2.676 11209064 PMC14647

[B29] Rossi S. Hallett M. Rossini P. M. Pascual-Leone A. (2009). Safety, ethical considerations and application guidelines for the use of transcranial magnetic stimulation in clinical practice and research. Clin. Neurophysiol. 120, 2008–2039. 10.1016/j.clinph.2009.08.016 19833552 PMC3260536

[B30] Silvanto J. Muggleton N. G. Cowey A. Walsh V. (2007). Neural adaptation reveals state-dependent effects of transcranial magnetic stimulation. Eur. J. Neurosci. 25, 1874–1881. 10.1111/j.1460-9568.2007.05440.x 17408427

[B31] Silvanto J. Muggleton N. Walsh V. (2008). State-dependency in brain stimulation studies of perception and cognition. Trends Cogn. Sci. 12, 447–454. 10.1016/j.tics.2008.09.004 18951833

[B41] Sporns O. (2013). Network attributes for segregation and integration in the human brain. Curr. Opin. Neurobiol. 23, 162–171. 10.1016/j.conb.2012.11.015 23294553

[B32] Spreng R. N. Sepulcre J. Turner G. R. Stevens W. D. Schacter D. L. (2013). Intrinsic architecture underlying the relations among the default, dorsal attention and frontoparietal control networks of the human brain. J. Cogn. Neurosci. 25, 74–86. 10.1162/jocn_a_00281 22905821 PMC3816715

[B33] Szczepanski S. M. Kastner S. (2013). Shifting attentional priorities: control of spatial attention through hemispheric competition. J. Neurosci. 33, 5411–5421. 10.1523/jneurosci.4089-12.2013 23516306 PMC3651512

[B34] Wagner T. Rushmore J. Eden U. Valero-Cabre A. (2009). Biophysical foundations underlying TMS: setting the stage for an effective use of neurostimulation in the cognitive neurosciences. Cortex 45, 1025–1034. 10.1016/j.cortex.2008.10.002 19027896 PMC3417820

[B35] Walsh V. Ellison A. Battelli L. Cowey A. (1998). Task-specific impairments and enhancements induced by magnetic stimulation of human visual area V5. Proc. Biol. Sci. 265, 537–543. 10.1098/rspb.1998.0328 9569672 PMC1688918

[B36] Walsh V. Pascual-Leone A. Kosslyn S. M. (2003). Transcranial Magnetic Stimulation: A Neurochronometrics of Mind. Cambridge, MA: MIT press.

[B37] Weissman D. H. Roberts K. C. Visscher K. M. Woldorff M. G. (2006). The neural bases of momentary lapses in attention. Nat. Neurosci. 9, 971–978. 10.1038/nn1727 16767087

[B38] Wojciulik E. Kanwisher N. (1999). The generality of parietal involvement in visual attention. Neuron 23, 747–764. 10.1016/s0896-6273(01)80033-7 10482241

[B39] Wokke M. E. Scholte H. S. Lamme V. A. (2014). Opposing dorsal/ventral stream dynamics during figure-ground segregation. J. Cogn. Neurosci. 26, 365–379. 10.1162/jocn_a_00497 24116840

